# Supplementary material for: Modulation of Pore Opening of Eukaryotic Sodium Channels by π-Helices in S6
Source: J Phys Chem Lett. 2023 Jun 21;14(25):5876–81. doi: 10.1021/acs.jpclett.3c00803 (PMC10316397; doi:10.1021/acs.jpclett.3c00803)
Supplement: Supplementary file 2 — jz3c00803_si_002.pdf [file jz3c00803_si_002.pdf]

jz-2023-00803k.R1

Name: Peer Review Information for "Modulation of Pore Opening of Eukaryotic Sodium Channels by π-Helices in S6"

First Round of Reviewer Comments

Reviewer: 1

Comments to the Author

Choudhury and Delemotte presented a very interesting study that probed the conductance state of the eukaryotic Nav channel using molecular dynamics simulations. Their study builds on their previous work on the bacterial Nav channel. They showed that a pi-helix stretch in S6 of the eukaryotic Nav channel has a strong effect on pore hydration and cation conduction. This work will be of significant interest to biophysicists studying ion channels and computational biologists using similar techniques.

To improve the clarity of the work, I have some suggestions and questions:

1. The authors should consider illustrating the location of the short pi-helix in figure 1. This information will be particularly useful for readers who are not familiar with Nav channels.

2. In Figures 2E and 3C, the authors compare their simulated conductances with experimental values indicated by dashed lines. The authors should describe this information more carefully in their figure legends, and they should cite the source of the experimental values in the legends as well. In Fig. 3C, the Y-axes label is missing.

3. In Figure 2C, the authors should provide more information about the models shown. Are they the end-snapshot of the respective simulations? The information is missing, and the illustration of this figure panel is not very helpful for understanding the corresponding section in the manuscript ("This could be attributed to the presence of hydrophobic residues facing the gate, namely a tetrad at the level of I409/F937/I1468/V1766 and another one one helical turn below at 413/L941/I1472/I1770 (Figure 2C)").

4. In the introduction of the manuscript, the authors stated that "The DI, DII, and DIV S6 have been captured both with a fully α-helical content and with a short π-helix stretch below the selectivity filter (3,18-20). The DIII S6 helix, on the other hand, has always been captured containing a short π-helix across the different conformations of Nav channels (3-20)". What is the maximum and minimum number of pi-helices of different subunits in experimentally resolved structures? Are there any experimental evidences that the number of pi-helices could be correlated with the conductance?

5. What are the modulation factors for the pi-helix population, and are they correlated with other structural features in Nav channels?

Reviewer: 2

Comments to the Author

This is a very interesting paper that used the recently proposed open structure of Nav1.5 as a basis to examine how the presence of pi helices in different pore forming domains of the channel influence the ion current. By studying the influence of pi helices in different combination of domains they are able to prose a structural model for the different non-conducting, sub-conducting and open states of the channel. While the evidence is not yet conclusive, I believe this manuscript provides a very useful hypothesis that has deep mechanistic insight.

The authors are careful in the language they use to not over-claim their conclusions – using terms such as 'suggest' and 'hypothesize' which is appropriate given some of the (unavoidable) uncertainties in the study. I believe that some of these uncertainties could be made more explicit. For example – the starting structure is proposed to represent an open state of the channel as stated int eh structural paper. However, it is always challenging to ascribe whether it is the exact physiological open state given the introduced mutations, and microscopy conditions. Perhaps more importantly, as the authors acknowledge, the pore collapses in MD simulations if it is not retrained to the open state indicating that something may be missing from the simulation model or the structure itself. Similarly and more significantly, the simulations of the different pi helix models are restrained around the starting coordinates. So if the model is not correct then it will not be able to relax in the MD simulations and may influence hydration and ion permeation.

I don't think these are fatal flaws given that this manuscript is presenting a very interesting hypothesis. I simply recommend that some of these uncertainties be explicitly described in the paper – perhaps in the concluding discussion,

There are errors in the references – for example the reference for the critical recent Nav1.5 structure is actually given as (12) the 2017 NavPas structure. All the references in the manuscript need to be carefully checked.for errors.

There is some confusion with the section discussing recent structure of Nav1.7 featuring a π-helix in DIV-S6 (line 34 page 7). I assume the authors are referring to citation 17 (a citation here would be handy). In this paragraph, what is the 3pi_d4 model? Figure S11 is also confusing as the axis/legend states the 3pi_d1_d3_d4 model was compared but the figure caption states it is the 3pi_d2_d3_d4.

Line 55 page 3… describes how all eukaryotic Nav structures have been captured with at least one out of the four S6 helices containing pi-helix. Just curious as to whether the authors think an all-alpha helix model represents feasible conformation (perhaps a non-conductive resting state) and maybe this model is worth testing also ?

Fig 3c is missing y axis label.

Reviewer: 3

Comments to the Author

This manuscript builds on previous work from the same group exploring the role of potential Pi helices in voltage-gated sodium channels. In this work, they explore how the presence of different combinations of pi helices in the S6 helix in the eukaryotic Nav1.5 channel affect predicted conductance. The work is entirely theoretical and presents an interesting hypothesis is gaining some traction as similar pi helices have been observed in TRP channels as well.

The manuscript is well-written and easy to follow. However, I do have some points that I would like the authors to address:-

1. The main issue for me is that all of the results are presented on the basis of relatively short simulations on a heavily constrained protein backbone. I understand the reasons for that, but it is nevertheless concerning that even the initial “native” structures undergo hydrophobic collapse (suggested the cryo-EM models are not optimal, or there is a force-field problem). Have the authors tried at least for the cryo-EM model to use a slow relaxation scheme that this model can be stable? If not, what about the d4-pi model – that presumably is the most hydrated- one might expect that to be stable without restraints on as the presence of the hydrating water will help to maintain that state.

2. This may be beyond the scope of their work, but they don't explain how the alpha-pi or pi-alpha transitions might be coupled to voltage sensor activation. Considering that they are using an open state as template, with the voltage sensors already activated, it is not clear to me what might trigger the transitions. Similarly, they mention the IFM motif and its role in the introduction and in the final paragraph, but don't try to connect it to what they are describing for the pi helices. What is the order of events in fast inactivation? VSD 4 activation first, then pi to alpha transition, than IFM binding? or IFM binding before helical transition?

3. They used Catterall's open state rat Nav1.5 structure (PDB 7FBS) as their template. As with all eukaryotic structures published to date, the N- and C-terminal domains as well as much of the intracellular loops between DI/DII and DII/DIII is missing. 7FBS also lacks a few residues in the S3-S4 linkers of DI and DII, and in the ECD of DI. One important aspect of obtaining the open state structure was the mutation of the IFM motif to QQQ, which disrupted fast inactivation. The region containing the IFM/QQQ sequence is also not resolved. The method section does not mention how they handled these gaps. I'm assuming they modelled short missing bits in and capped the residues surrounding the missing intracellular loops, but they don't say. Because they mention fast inactivation a number of times, mentioning how they handled the IFM/QQQ in their simulations would be helpful.

4. Related to the above, they don't provide links to their homology models. It would be great to release these. Providing a Zenodo share link or similar would be great.

5. I didn't see any analyses of statistical significance but I think that might be useful information here. And related to ion conductance - In Fig 3C, the y axis label is missing, and the lower half of the error bar for 3pi_d2_d3_d4 can't be seen.

6. Another issue is that the ends of the PMFs in S10 do not show convergence because the ends each profile should be at the same value – the plots need extending in the pore dimension until the value

outside of the pore is the same otherwise the PMFs are rather meaningless. Besides that – as schematic of “where we are” (see below) would be helpful.

7. I would have like to have seen a figure that shows the difference effects of the different pi helices on what lines the pore – that would be super-interesting.

8. Please use/develop a schematic that allows us to interpret where we actually are in the SI plots. Something like Figure 1D (or even better a cut-away that shows the inner-pore surface with the box coordinates super-imposed.

9. I'd really like their water number density plots in 2B and 3B to have the same y axis as their free energy plots in 2D and 4D to make comparisons easier. I think the colours in figure 3 are suboptimal. I'm not colourblind and I still struggled identifying 4pi, 3pi_d1_d2_d3, and 3pi_d1_d3_d4. This was especially hard in the water number density plot because of the overlapping shaded areas. These figures also could benefit from a coordinate axis superposed onto a figure like 1D, but with more residues highlighted, so we could get a better idea of where in the pore things are.

10. On page 7, line 36-ish, they start talking about a 3pi_d4 model that isn't mentioned anywhere else in the paper. I'm assuming they mean either the 3pi_d2_d3_d4, the 3pi_d1_d3_d4, or both, but it's not clear to me.

11. Typos

a. Page 5, line 17ish: "at 413/L941/I1472/I1770", residue before 413 is missing

b. Page 7, line 42ish: "We observed no effect on pore hydration (FIgure S11B)" Figure has capital I

c. Page 8, line 14ish: "surmise that an π to □-helix" should be a pi to alpha.

Author's Response to Peer Review Comments:

Reviewer: 1

Recommendation: This paper is publishable subject to minor revisions noted. Further review is not needed.

Comments:
Choudhury and Delemotte presented a very interesting study that probed the conductance state of the eukaryotic Nav channel using molecular dynamics simulations. Their study builds on their previous work on the bacterial Nav channel. They showed that a pi-helix stretch in S6 of the eukaryotic Nav channel has a strong effect on pore hydration and cation conduction. This work will be of significant interest to biophysicists studying ion channels and computational biologists using similar techniques.

We appreciate the reviewer's evaluation of our work and are glad that they think this work will be of interest in our community.

To improve the clarity of the work, I have some suggestions and questions:
1. The authors should consider illustrating the location of the short pi-helix in figure 1. This information will be particularly useful for readers who are not familiar with Nav channels.
Thank you for the suggestion, we have now highlighted the location of the pi helix in figure 1D.

2. In Figures 2E and 3C, the authors compare their simulated conductances with experimental values indicated by dashed lines. The authors should describe this information more carefully in their figure legends, and they should cite the source of the experimental values in the legends as well. In Fig. 3C, the Y-axes label is missing.
We have now added this information in the respective figure legends and added a citation to the source of experimental values. We have also added the missing y-axis label in figure 3C.

3. In Figure 2C, the authors should provide more information about the models shown. Are they the end-snapshot of the respective simulations? The information is missing, and the illustration of this figure panel is not very helpful for understanding the corresponding section in the manuscript ("This could be attributed to the presence of hydrophobic residues facing the gate, namely a tetrad at the level of I409/F937/I1468/V1766 and another one one helical turn below at 413/L941/I1472/I1770 (Figure 2C)").
Thank you for your comment. We have now added the time at which the snapshots were taken. We had not referenced the figures properly, thank you for pointing out this oversight, which has now been corrected.

The idea behind figure 2C is that an alpha/pi helix transition in the DII-S6 does not change the hydrophobicity of the pore-facing residues around the activation gate, while an alpha/pi helix transition in the DIV-S6 causes a significant change in the hydrophobicity of the pore facing residues. In addition, an alpha/pi helix transition in the DI-S6 causes a slight change in the hydrophobicity of the residues facing the pore around the activation gate. Based on the nature

of the residue facing the activation gate, the pore hydration/ion permeation/free energy of the ion permeation is affected and hence the contribution of the different S6 segments is different.

4. In the introduction of the manuscript, the authors stated that “The DI, DII, and DIV S6 have been captured both with a fully α-helical content and with a short π-helix stretch below the selectivity filter (3,18-20). The DIII S6 helix, on the other hand, has always been captured containing a short π-helix across the different conformations of Nav channels (3-20)”. What is the maximum and minimum number of pi-helices of different subunits in experimentally resolved structures? Are there any experimental evidences that the number of pi-helices could be correlated with the conductance?
There is only one pi helix in the S6 per subunit. This observation is based on the cryo-EM structures. Currently, there is no experimental evidence that shows that the number of pi-helices and the conductance are correlated.

5. What are the modulation factors for the pi-helix population, and are they correlated with other structural features in Nav channels?1
Experimental structures have revealed that there are two modulation factors for the pi-helix populations:

- Conformation of the VSD (whether the VSD is activated or deactivated) as shown in ref 17,18. Structural studies have shown that a deactivated VSD in subunit I and II resulted in pi helix in all the pore lining helices. Structural comparison with the structures with activated VSDs showed that this process is mediated through the S5 helices of adjacent subunit which is the non-canonical pathway.
- Binding of certain drugs as shown in ref 19. Drug binding in the central cavity of the pore also facilitated an alpha to pi transition in different pore lining helices. However, the exact mechanism of this modulation is not known.

These observations were made based on structural comparisons and there are no functional experiments to support these claims. Thus, we chose to not discuss this in the current manuscript.

Reviewer: 2

Recommendation: This paper is publishable subject to minor revisions noted. Further review is not needed.

Comments:
This is a very interesting paper that used the recently proposed open structure of Nav1.5 as a basis to examine how the presence of pi helices in different pore forming domains of the channel influence the ion current. By studying the influence of pi helices in different combination of domains they are able to prose a structural model for the different non-conducting, sub-conducting and open states of the channel. While the evidence is not yet conclusive, I believe this manuscript provides a very useful hypothesis that has deep mechanistic insight.

We thank the reviewer for evaluating our manuscript and appreciate their comments. Indeed we also agree that our hypothesis warrants further work using complementary techniques!

The authors are careful in the language they use to not over-claim their conclusions – using terms such as ‘suggest’ and ‘hypothesize’ which is appropriate given some of the (unavoidable) uncertainties in the study. I believe that some of these uncertainties could be made more explicit. For example – the starting structure is proposed to represent an open state of the channel as stated in the structural paper. However, it is always challenging to ascribe whether it is the exact physiological open state given the introduced mutations, and microscopy conditions. Perhaps more importantly, as the authors acknowledge, the pore collapses in MD simulations if it is not retrained to the open state indicating that something may be missing from the simulation model or the structure itself. Similarly and more significantly, the simulations of the different pi helix models are restrained around the starting coordinates. So if the model is not correct then it will not be able to relax in the MD simulations and may influence hydration and ion permeation.
This is a very important point that we had tried to highlight in the original submission. In particular, we (and others) believe that there are stabilizing factors in the unresolved intracellular domain that maintain the pore in an expanded conformation. In absence of such domains, we have chosen to apply position restraints to evaluate the permeation and hydration properties of the starting model.

I don’t think these are fatal flaws given that this manuscript is presenting a very interesting hypothesis. I simply recommend that some of these uncertainties be explicitly described in the paper – perhaps in the concluding discussion,
Thank you for your comment. We agree that this caveat could be repeated once more in the conclusion section. We have thus added the following sentence - ‘Although the simulations in this study were performed under restraints, the results presented in this study reveal an important role of π-helix on pore hydration/ion permeation given an expanded pore conformation.’ in the concluding discussions.

There are errors in the references – for example the reference for the critical recent Nav1.5 structure is actually given as (12) the 2017 NavPas structure. All the references in the manuscript need to be carefully checked.for errors.
Thank you for pointing this out. We have now corrected the references.

There is some confusion with the section discussing recent structure of Nav1.7 featuring a π-helix in DIV-S6 (line 34 page 7). I assume the authors are referring to citation 17 (a citation here would be handy). In this paragraph, what is the 3pi_d4 model? Figure S11 is also confusing as the axis/legend states the 3pi_d1_d3_d4 model was compared but the figure caption states it is the 3pi_d2_d3_d4.
Thank you for pointing these out, we have now added the reference. Sorry about the mistake, this model is actually the 3pi_d1_d3_d4 model, we have now made corrections.

Line 55 page 3… describes how all eukaryotic Nav structures have been captured with at least one out of the four S6 helices containing pi-helix. Just curious as to whether the authors think an

all-alpha helix model represents feasible conformation (perhaps a non-conductive resting state) and maybe this model is worth testing also ?
Thank you for this interesting question. An answer to this would be purely speculative. Since there is no experimentally resolved structure of DIII-S6 in an alpha helical conformation we decided not to pursue such a model. Indeed, the unavailability of an experimental models of DIII-S6 with an alpha helical conformation made modeling such a state more uncertain, and we thus decided to steer away from testing such a model.

Fig 3c is missing y axis label.
Thank you for pointing this out. We have now added the missing label.

Reviewer: 3

"This manuscript builds on previous work from the same group exploring the role of potential Pi helices in voltage-gated sodium channels. In this work, they explore how the presence of different combinations of pi helices in the S6 helix in the eukaryotic Nav1.5 channel affect predicted conductance. The work is entirely theoretical and presents an interesting hypothesis is gaining some traction as similar pi helices have been observed in TRP channels as well.
We thank the reviewer for evaluating our manuscript and appreciate the comments.

The manuscript is well-written and easy to follow. However, I do have some points that I would like the authors to address:-

1. The main issue for me is that all of the results are presented on the basis of relatively short simulations on a heavily constrained protein backbone. I understand the reasons for that, but it is nevertheless concerning that even the initial “native” structures undergo hydrophobic collapse (suggested the cryo-EM models are not optimal, or there is a force-field problem). Have the authors tried at least for the cryo-EM model to use a slow relaxation scheme that this model can be stable? If not, what about the d4-pi model – that presumably is the most hydrated- one might expect that to be stable without restraints on as the presence of the hydrating water will help to maintain that state.
Thank you for your comment, this was also raised by reviewer 2, see answer above. To answer more concretely this reviewer’s point, we did try to gradually release the restraints after 30 ns of restrained simulations for the original cryo-EM model. However, upon releasing the restraints the pore collapsed. The same holds for the d4-pi models, which could be anticipated to be even more stable in the open state given their increased hydration.

Note that there are intracellular linkers between the S6 and S1 helices of all the domains and that those are difficult to resolve experimentally, presumably due to their disordering. We thus hypothesize that the absence of these intracellular linkers causes a pore collapse. This hypothesis is supported by our earlier studies of bacterial channels (dois 10.1016/j.bpj.2021.12.010 and 10.1085/jgp.202213214), where we observed pore collapse

upon removal of the intracellular domains. In the bacterial NavAb, in absence of intracellular domains, we had also performed a slow equilibration akin to what this reviewer suggested and observed pore collapse.

The authors of the experimental open state of Nav1.5 paper (ref 15 in SI) also performed MD simulations and observed pore collapse. We acknowledge that this could be made worse by force field limitations but given that other open channels can be modeled using similar setups and force fields, however we are more inclined to believe that it is the quality of the experimental model that is in question.

2. This may be beyond the scope of their work, but they don't explain how the alpha-pi or pi-alpha transitions might be coupled to voltage sensor activation. Considering that they are using an open state as template, with the voltage sensors already activated, it is not clear to me what might trigger the transitions. Similarly, they mention the IFM motif and its role in the introduction and in the final paragraph, but don't try to connect it to what they are describing for the pi helices. What is the order of events in fast inactivation? VSD 4 activation first, then pi to alpha transition, than IFM binding? or IFM binding before helical transition?
Thank you for you question. We also can only speculate about what could trigger the transition. It is very difficult to say at the moment as we do not have a resting state model of Nav1.5. There are structural studies wherein comparing the closed inactivated and the fast-inactivated states has revealed a non-canonical VSD-PD coupling pathway that might enable this transition.

Based on the structures so far, we have discussed the order of events in fast inactivation in the introduction, namely that fast inactivation occurs after DIV-VSD activation which allows the IFM to bind. There are experimental structures of fast inactivated states (IFM particle docked in its binding site) that have a pi-helix in DIV-S6 and there are other fast inactivated structures that feature an alpha-helix in DIV-S6. It is thus difficult to connect IFM binding and secondary structure transitions in S6 and in absence of new data, we have chosen to refrain from discussing the role of IFM binding in S6 transitions. We obviously agree that this is an important and interesting question and look forward to learning more about it through our own and others' work.

3. They used Catterall's open state rat Nav1.5 structure (PDB 7FBS) as their template. As with all eukaryotic structures published to date, the N- and C-terminal domains as well as much of the intracellular loops between DI/DII and DII/DIII is missing. 7FBS also lacks a few residues in the S3-S4 linkers of DI and DII, and in the ECD of DI. One important aspect of obtaining the open state structure was the mutation of the IFM motif to QQQ, which disrupted fast inactivation. The region containing the IFM/QQQ sequence is also not resolved. The method section does not mention how they handled these gaps. I'm assuming they modelled short missing bits in and capped the residues surrounding the missing intracellular loops, but they don't say. Because they mention fast inactivation a number of times, mentioning how they handled the IFM/QQQ in their simulations would be helpful.

Thank you for bringing this up. We did miss to mention this in the methods section. Since we are interested in the open state in this manuscript, we decided to remove the DIII-DIV linker for the following reasons:

- In the open state the IFM is not supposed to be bound
- Since the IFM particle is not well resolved in the experimental structure, we avoided arbitrary modeling choices.

The missing residues in the S3-S4 loop and the ECD of DI were modelled using CHARMM-GUI and the residues surrounding the missing intracellular loops were capped. We agree that this was an oversight and have modified the methods section to reflect this.

4. Related to the above, they don't provide links to their homology models. It would be great to release these. Providing a Zenodo share link or similar would be great.
Thank you for bringing this up. We have now uploaded all the data (including the systems for the homology models) related to OSF and shared the OSF link.

5. I didn't see any analyses of statistical significance but I think that might be useful information here. And related to ion conductance - In Fig 3C, the y axis label is missing, and the lower half of the error bar for 3pi_d2_d3_d4 can't be seen.
Thank you for highlighting this. We have now performed a two-sample t-test to analyze the statistical significance of the ion conductances calculated in fig 2E and 3C and highlighted this in the respective plots. We have added the missing label in Fig 3C. We have changed the colors of the error bars in Fig 3C to improve visibility.

6. Another issue is that the ends of the PMFs in S10 do not show convergence because the ends each profile should be at the same value – the plots need extending in the pore dimension until the value outside of the pore is the same otherwise the PMFs are rather meaningless. Besides that – as schematic of “where we are” (see below) would be helpful.
The ends of the profiles do not show the same value because one end is inside the protein and another end is in the solvent. We did not extend the y-axis of the free energy profile beyond the selectivity filter for the following reasons:

- We mainly modify the activation gate region and there is no modification around the selectivity filter region. Thus, we are not particularly interested in the free energy around around the selectivity and are only interested in the free energy around the pi helix site .
- Converging the free energy in the selectivity filter was quite challenging.

We have added a translucent version of Fig1D in all the water number density and ion permeation free energy profiles to show ‘where we are’ in the pore.

7. I would have like to have seen a figure that shows the difference effects of the different pi helices on what lines the pore – that would be super-interesting.
Figure 2C highlights what residues are lining the pore upon introducing pi helices in different pore lining helices. We hope that we have interpreted properly this reviewer’s comment.

8. Please use/develop a schematic that allows us to interpret where we actually are in the SI plots. Something like Figure 1D (or even better a cut-away that shows the inner-pore surface with the box coordinates super-imposed.
Thank you for your comment. We have now shown a translucent version of figure 1D in all the main figure and SI plots to improve the understanding of where 'we are' actually in the pore.

9. I'd really like their water number density plots in 2B and 3B to have the same y axis as their free energy plots in 2D and 4D to make comparisons easier. I think the colours in figure 3 are suboptimal. I'm not colourblind and I still struggled identifying 4pi, 3pi_d1_d2_d3, and 3pi_d1_d3_d4. This was especially hard in the water number density plot because of the overlapping shaded areas. These figures also could benefit from a coordinate axis superposed onto a figure like 1D, but with more residues highlighted, so we could get a better idea of where in the pore things are.
Thank you for your suggestion. Since the water number density plots are calculated using CHAP and the free energy is projected on a CV as defined in the methods section, it was challenging for us to have 'exactly' the same axis for both of them. We have however now displayed figure 1D in the background of all these plots to will enable comparisons.
We have also changed the color scheme in Figure 3 to make it more interpretable and superimposed figure 1D to ease comparisons. Showing residues really makes the figures difficult to digest and we have thus chosen to avoid that. Instead, the residues around the barriers regions (for 2pi_d1_d3 and 3pi_d1_d2_d3 model as they had the highest free energy barriers) are labeled in the free energy plots.

10. On page 7, line 36-ish, they start talking about a 3pi_d4 model that isn't mentioned anywhere else in the paper. I'm assuming they mean either the 3pi_d2_d3_d4, the 3pi_d1_d3_d4, or both, but it's not clear to me.
Thank you for pointing this out. This was a typo. We were referring to the 3pi_d1_d3_d4 model, and this has now been corrected.

11. Typos

a. Page 5, line 17ish: "at 413/L941/I1472/I1770", residue before 413 is missing

b. Page 7, line 42ish: "We observed no effect on pore hydration (FIgure S11B)" Figure has capital I

c. Page 8, line 14ish: "surmise that an π to □□-helix" should be a pi to alpha."
Thank you for pointing out these typos. We have now corrected them.
